# High-efficiency Nebulizers Reduce Albuterol Administration Time in the Emergency Department

**DOI:** 10.5811/westjem.53169

**Published:** 2026-10-18

**Authors:** James McCue, Darin Craft, Wade Veneman, Rawnica Ruegner, Brian Chinnock, Jannet Castaneda, Jonathan Broadhurst, Alec Chan-Golston, Yueqi Yan, Danielle Campagne

**Affiliations:** * University of California San Francisco, Department of Emergency Medicine, Fresno, California; † Community Regional Medical Center, Department of Emergency Medicine, Fresno, California; ‡ Community Regional Medical Center, Department of Respiratory Care, Fresno, California; § University of California Merced, Health Sciences Research Institute, Merced, California

## Abstract

**Introduction:**

Asthma exacerbations commonly require nebulized albuterol in the emergency department (ED). High-efficiency devices (vibrating mesh, breath-enhanced/actuated) may shorten treatment time compared with traditional jet nebulizers. We compared four commonly used nebulizer devices on treatment time and ED outcomes.

**Methods:**

We conducted a prospective observational study of a convenience sample of patients at a high-volume, urban Level I trauma center ED (February 2019–March 2023; paused during COVID-19). Patients ≥ 3 years of age receiving clinician-ordered nebulized albuterol were eligible. Exclusions included patients with a history of chronic obstructive pulmonary disease or congestive heart failure, inability to follow commands or hold the device, and need for positive-pressure ventilation. Respiratory care practitioners used one of four devices already in use in this department: Westmed miniHEART Hi-Flo jet (Mini), Aerogen Ultra (vibrating mesh), Monaghan AeroEclipse II (breath-actuated), or Salter Labs NebuTech (breath-enhanced). The Mini group received a single 15-mg dose; others received 5-mg increments up to 15 mg with reassessment. Our primary outcome measure was the shortest time to complete delivery of the planned albuterol dose to the patient. Secondary outcomes included hospital admission and changes in asthma security scores and peak expiratory flow. We used analysis of variance post-hoc tests and adjusted linear/logistic regression. Both males and females were included; sex-stratified analyses were not prespecified.

**Results:**

We enrolled 296 patients (Mini, n = 90; Aerogen, n = 67; AeroEclipse, n = 65; NebuTech, n = 74), 78 (26.3%) of whom were pediatric patients 3–18 years of age (Mini, n = 22; Aerogen, n = 20; AeroEclipse, n = 14, and NebuTech, n = 22). Time to complete albuterol differed by device, with mean treatment times in minutes (95% CI): Mini, 60.6 [55.9–65.3]; Aerogen, 12.3 [10.9–13.7]; AeroEclipse, 8.7 [7.7–9.7]; and NebuTech, 7.9 [7.0–8.8]. Adjusted models showed 82%, 88%, and 88% relative reductions vs Mini (all *P* < .01). Admission rates did not differ in total percentage (Mini, 11.1 [10]; Aerogen, 14.9 [10]; AeroEclipse, 16.9 [11]; and NebuTech, 6.8 [5] (all *P* > .05). Adults had lower odds of admission than pediatrics (odds ratio 0.38; 95% CI, 0.18–0.79; *P* = .01), with a total of 36 (12.1%) patients requiring admission, two pediatric. Asthma severity and improvements in peak expiratory flow were similar across devices, with mean post-treatment values (asthma severity score and peak expiratory flow): Mini, 5.5 and 301.1; Aerogen, 5.6 and 292.7; AeroEclipse, 5.9 and 248.5; and NebuTech, 5.7 and 301.1 (all *P* > .01).

**Conclusion:**

In a real-world ED cohort, vibrating-mesh and breath-enhanced/actuated nebulizers delivered albuterol > 80% faster than a traditional jet device, potentially saving at least 45 minutes per treatment per patient, without differences in admission rates or clinical response. Adopting faster devices may improve workflow and throughput without compromising patient outcomes.

## INTRODUCTION

Asthma exacerbations are a common presentation to the emergency department (ED). The fundamental physiology of asthma in both pediatric and adult patients is chronic inflammation of the lower airways causing narrowing of the airways through a combination of swelling, secretions and muscle constriction of the bronchi.^1^ According to the American Lung Association State of the Air Report 2016, over 550,000 people in the San Joaquin Valley in California have asthma. While studies show that asthma is a heterogenous disease with more patient-focused treatments being developed, traditional asthma exacerbation treatments for both pediatric and adult patients in the ED include steroids and nebulizer treatments of albuterol, a short-acting beta-agonist, which can be an hour-long treatment in more moderate-severe exacerbations. Inhalers may also be considered in the treatment of asthma in the ED; however, patient compliance can be variable due to the coordination needed to time one’s breath with the medication administration.

Many nebulizer devices now on the market deliver albuterol in minutes and potentially more reliably than an inhaler.^2^,^3^ We included four nebulizer models in this study: the Westmed Inc miniHEART Hi-Flo Jet Nebulizer (Mini); the Monaghan AeroEclipse II Breath-Actuated Nebulizer (AeroEclipse); the Salter Labs NebuTech High-Density Nebulizer (NebuTech); and the Aerogen Ultra Nebulizer (Aerogen). These devices were selected because they are currently in use across various clinical settings within our hospital and have been prioritized by clinicians for purchase, ensuring that the study reflects the use of real-world equipment. Their inclusion ensured that the study captured the range of technologies most relevant to optimizing aerosol delivery in clinical practice. Although prior studies have evaluated the performance of these nebulizers individually,^4^,^5^ no direct head-to-head comparisons exist to identify which device is most effective for the rapid delivery of aerosolized medication.

The Mini produces aerosol continuously and can aerosolize up to 30 milliliters of medication and has a higher dead volume.^6^ The AeroEclipse generates aerosol only on inhalation, offers higher medication concentration, and has a high dead volume.^2^ The NebuTech stores aerosol in a reservoir and reduces atmospheric medication loss, although its dead volume remains high.^3^ The Aerogen, a handheld device, used in conjunction with the Aerogen Solo, is a vibrating-mesh nebulizer requiring no gas source (although one may be used); it is associated with a very low dead volume.^7^

## OBJECTIVE

In this study we aimed to evaluate and compare four high-efficiency nebulizer devices in terms of time to complete albuterol administration, and hospital admission rates among patients presenting with asthma exacerbations, improving asthma severity, and peak expiratory flow.

**Population Health Research Capsule**

What do we already know about this issue?

*Prior studies have evaluated the performance of nebulizers individually, but no direct head-to-head comparisons exist.*

What was the research question?

*We compared four nebulizer devices regarding time to complete albuterol administration, and hospital admission rates.*

What was the major finding of the study?

*Mean treatment times in minutes (95% CI): Mini, 60.6 (55.9–65.3); Aerogen, 12.3 (10.9–13.7; AeroEclipse, 8.7 (7.7–9.7), and NebuTech, 7.9 (7.0–8.8).*

How does this improve population health?

*Vibrating-mesh and breath-enhanced/actuated nebulizers deliver albuterol > 80% faster than traditional jet devices, without changes in admission rates or clinical response.*

## METHODS

### Study Design

This was a prospective, observational study of ED patients presenting with asthma exacerbations requiring albuterol treatment via nebulizer. All nebulizer devices were purchased through a non-industry grant and were already in routine use. These devices were identified by the respiratory therapy department in our hospital and are actively used by the respiratory therapy team across various departments within the institution, allowing the study to reflect real-world clinical practice patterns and procurement considerations. The manufacturers provided no financial support, equipment, or input regarding study design, analysis, writing, or publication decisions.

Approximate hospital acquisition costs at the time of study were as follows: Mini, ≈ $9.36 per unit; Aerogen, ≈ $48.57 per unit; AeroEclipse, ≈ $4.57 per unit; and NebuTech, ≈ $2.54 per unit. The study took place between February 2019–March 2023 with a 13-month pause due to the COVID-19 pandemic (last patient enrolled on March 12, 2020, and study resumed on April 13, 2022). The pause secondary to the COVID-19 pandemic did not change nebulizer use pre- versus post-pandemic, due to patients needing rooms or designated respiratory areas prior to nebulizer administration throughout the course of the study. We obtained study approval from the institutional review board, and the study was supported by a non-industry institutional research grant.

### Setting

This study was conducted in the ED of a busy academic Level I trauma center and tertiary-care hospital in a large metropolitan area with ED volumes > 120,000 patient visits per year.

### Selection of Participants

Eligible participants presented with reactive airway disease, shortness of breath, cough, or other respiratory complaint in which nebulized albuterol was ordered. We included in the study patients ≥ 3 years of age. Patients were excluded if they had a known history of chronic obstructive pulmonary disease or congestive heart failure, were unable to follow instructions or hold nebulizer devices, or they required positive-pressure ventilation (eg, bilevel positive airway pressure, intubation, or high-flow oxygen). The enrollment process is summarized in Figure 1.

### Study Protocol and Interventions

Patients were screened for inclusion by the respiratory care practitioner (RCP). For pediatric patients, the severity of asthma was determined by the Pediatric Asthma Severity Score (PASS). For adult patients, the severity of asthma was determined by our modified adult asthma severity score (mAASS), which was adapted from PASS, adjusting respiratory rate and oxygenation status. Pre-treatment peak expiratory flow rates were also recorded. The RCP then delivered the clinician-ordered albuterol, selecting a nebulizer device from the research bin in the ED supply closet, which was restocked by our research coordinator weekly. Treatments were administered per device protocol, and repeated doses or steroid administration were left to the clinician’s discretion. 8,9

The study focused solely on nebulizer efficiency, not medication dose standardization. For the control group (Mini), a single 15-mg albuterol dose was administered. We used this device as the control group as this was the local practice pattern. For the other three nebulizer devices, we administered an albuterol 5-mg dose, with the RCP assessing the patient after each 5-mg dose and redosing up to a total of 15. mg. The RCPs remained with the patients during their entire treatment if they were using the Aerogen, NebuTech or AeroEclipse, and for the Mini. They revaluated the patient every 15 minutes until the administration was complete. Once the patient completed their 5-, 10- or 15-mg dose, the RCP would notify the ordering clinician for re-evaluation.

### Methods of Measurement

We used separate pediatric and adult data collection forms. We documented the nebulizer type used, chief complaint, history of pulmonary disease, and disposition (admission versus discharge). Pre- and post-treatment PASS/mAASS and peak expiratory flow measurements were documented, as well as the dose of albuterol given.

### Outcome Measures

Our primary outcome measure was to identify which device could deliver albuterol to the patient in the shortest time. Our secondary outcome measures were to determine which device provided the greatest improvement to possibly decrease admission rates, while improving asthma severity and peak expiratory flow.

## RESULTS

We enrolled 296 patients in the study: 90 received treatment via the Mini, 67 via the Aerogen, 65 via the AeroEclipse, and 74 via the NebuTech. Demographic variables, including age, sex, and adult/pediatric, were comparable across groups, although the Mini and AeroEclipse devices were more frequently used in adults (*P* < .01; Table 1).

Significant differences were observed in albuterol administration time among the four nebulizer groups (*P* < .01, analysis of variance). The geometric mean administration time was 60.6 minutes (95% CI, 55.9–65.3) for the Mini; 12.3 minutes (10.9–13.7) for the Aerogen; 8.7 minutes (7.7–9.7) for the AeroEclipse; and 7.9 minutes (7.0–8.8) for the NebuTech. In fully adjusted linear regression models, compared to the Mini, the Aerogen, AeroEclipse, and NebuTech devices were associated with an 82% (75–87%), 88% (82–92%), and 88% (83–92%) reduction in administration time, respectively (all *P* < .01). Post-hoc pairwise comparisons revealed no significant difference between the AeroEclipse and NebuTech (*P* = 1.00), while both were significantly faster than the Aerogen (*P* < .01).

Admission rates did not differ significantly by nebulizer type (*P* = .10, χ^2^) as noted in Table 1, including in the pediatric population, of whom only two required admission (one each in the Aerogen and Nebutech study arms). In logistic regression models, odds of admission were not significantly different across devices. However, adults had significantly lower odds of admission than pediatric patients (odds ratio 0.38, 95% CI, 0.18–0.79, *P* = .01).

Neither peak expiratory flow improvement nor asthma severity score (mAASS/PASS) change differed significantly by device. The 95% CIs for both measures overlapped across all four groups, indicating comparable post-treatment improvements as shown in Figures 2 and 3.

## DISCUSSION

Our results demonstrated more rapid initiation and delivery of albuterol nebulizer treatment with the vibrating- mesh and breath-enhanced nebulizers than the jet nebulizer.^2^,^4^,^7^ This study presents a real-world comparison of four commonly used nebulizer devices in an academic ED, offering critical insight into their relative efficiency in delivering albuterol for asthma exacerbations. The Aerogen, AeroEclipse, and NebuTech devices significantly outperformed the traditional Mini nebulizer in terms of administration time, reducing it by > 80%. These improvements potentially have meaningful implications for clinical workflow and ED throughput, especially in high-volume or resource-limited settings.

We found substantial differences in delivery time, yet no statistically significant differences were found in clinical outcomes, including post-treatment peak expiratory flow, mAASS, or hospital admission rates. This finding suggests that while device efficiency may optimize workflow, it does not necessarily translate into improved short-term clinical outcomes or hospital admission rates. It also suggests that newer nebulizer technologies are more efficient and do not compromise patient care or safety. Interestingly, our analysis also showed that adult patients were less likely to be admitted, a finding that may reflect differing thresholds for inpatient care between pediatric and adult populations.

These results align with prior studies demonstrating that breath-actuated and vibrating-mesh technologies are timelier in aerosol drug delivery than continuous jet nebulizers. However, our study adds a novel contribution by evaluating these devices side-by-side in a live ED environment, capturing the operational impact of device selection on care delivery.

The RCPs expressed their preference for the NebuTech due to faster delivery time and their subjective evaluation that patients appeared better after treatment, in tandem with objective findings of improved peak epiratory flows post-treatment. They also reported that the Aerogen was the least favored nebulizer secondary to requiring both a power source and oxygen tank for medication administration, which can be difficult to find in a busy, urban ED. The data and experiences from this study have led our hospital system’s respiratory department to propose the use of the NebuTech nebulizer as the standard in our EDs.

## LIMITATIONS

This study has several limitations. First, it was conducted at a single center using a convenience sampling design, limiting generalizability. Second, nebulizer device choice was influenced by device availability, rather than per protocol, introducing potential selection bias. Third, although we controlled for key covariates in regression models, unmeasured confounding (eg, severity of asthma beyond initial scoring and patient comorbidities) could have influenced outcomes such as admission rates. Fourth, we used a modified PASS score for our adult patients; this score has not been validated in prior studies. Finally, medication dosing was not standardized across all devices, reflecting real-world practice but potentially affecting treatment times.

## CONCLUSION

In this prospective, observational study of albuterol delivery via nebulizers in asthma patients in the emergency department, the vibrating-mesh and breath-enhanced nebulizers significantly reduced administration time compared to the jet nebulization device. While clinical outcomes such as admission rates were similar across groups, the marked improvement in treatment time to initiation and completion suggests that using faster nebulizer technologies may optimize ED workflow and improve patient throughput. Future studies should evaluate the cost-effectiveness and long-term outcomes of implementing these devices in various healthcare settings.

## Supplementary Information



## Figures and Tables

**Figure 1:**
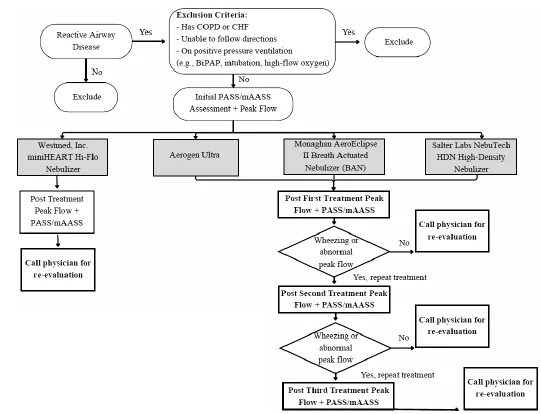
Flow diagram of study enrollment, exclusions, and treatment sequences for patients with asthma exacerbations who received nebulized albuterol. *PASS*, pediatric asthma severity score; *mAASS*, modified adult asthma severity score.

**Figure 2:**
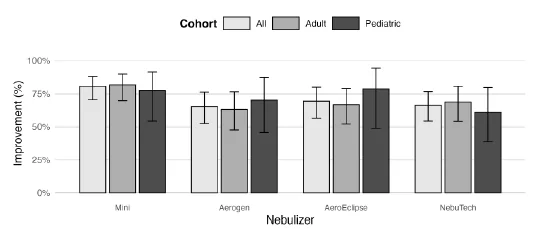
Bars indicate the percentage of paients demonstrating diminished asthma severity after treatment with one of four types of nebulizer devices. Error bars = 95% confidence intervals.

**Figure 3:**
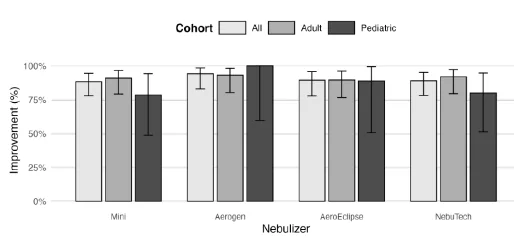
Bars indicate the proportion of patients whose peak expiratory flow increased after albuterol administration, based on type of nebulizer used. Error bars = 95% confidence intervals.

**Table 1:** Baseline demographic and clinical characteristics of emergency department patients receiving nebulized albuterol by device type in a prospective observational study.

	MiniHeart (*n* = 90)	Aerogen (*n* = 67)	AeroEclipse (*n* = 65)	Nebutech (*n* = 74)	*P* value
Demographics					
Age, mean (SD)	30.5 (16.6)	29.3 (18.7)	34.4 (17.2)	31.9 (18.8)	.38
Female, %, (*n*)	55.6 (50)	50.9 (34)	69.2 (45)	52.7 (39)	.14
Pediatric, % (*n*)	24.4 (22)	29.9 (20)	21.5 (14)	29.7 (22)	< .001
Clinical Outcome					
Length of stay, mean (SD)	15.3 (37.4)	13.3 (26.1)	22.0 (46.0)	9.5 (19.6)	.18
Admitted, % (*n*)	11.1 (10)	14.9 (10)	16.9 (11)	5 (6.8)	.10

Values are mean (SD) unless otherwise indicated. *P* values are from ANOVA for continuous variables and χ^2^ tests for categorical variables. Length of stay is reported in hours.

*SD*, standard deviation.
