# Supplementary material for: High-efficiency Nebulizers Reduce Albuterol Administration Time in the Emergency Department
Source: West J Emerg Med. 2026 Oct 18;27(6):1611–6. doi: 10.5811/westjem.53169 (PMC13762068; doi:10.5811/westjem.53169)

Appendix

Figure 4. Nebulizer devices compared in the study.

Photographs of the four nebulizer devices evaluated: Westmed miniHEART Hi-Flo (jet), Monaghan AeroEclipse II (breath-actuated), Aerogen Ultra (vibrating-mesh), and Salter Labs NebuTech (breath-enhanced).

Image sources: Tri-anim Health Services (miniHEART Hi-Flo, AeroEclipse II); Medline (NebuTech); HealthManagement.org (Aerogen Ultra).

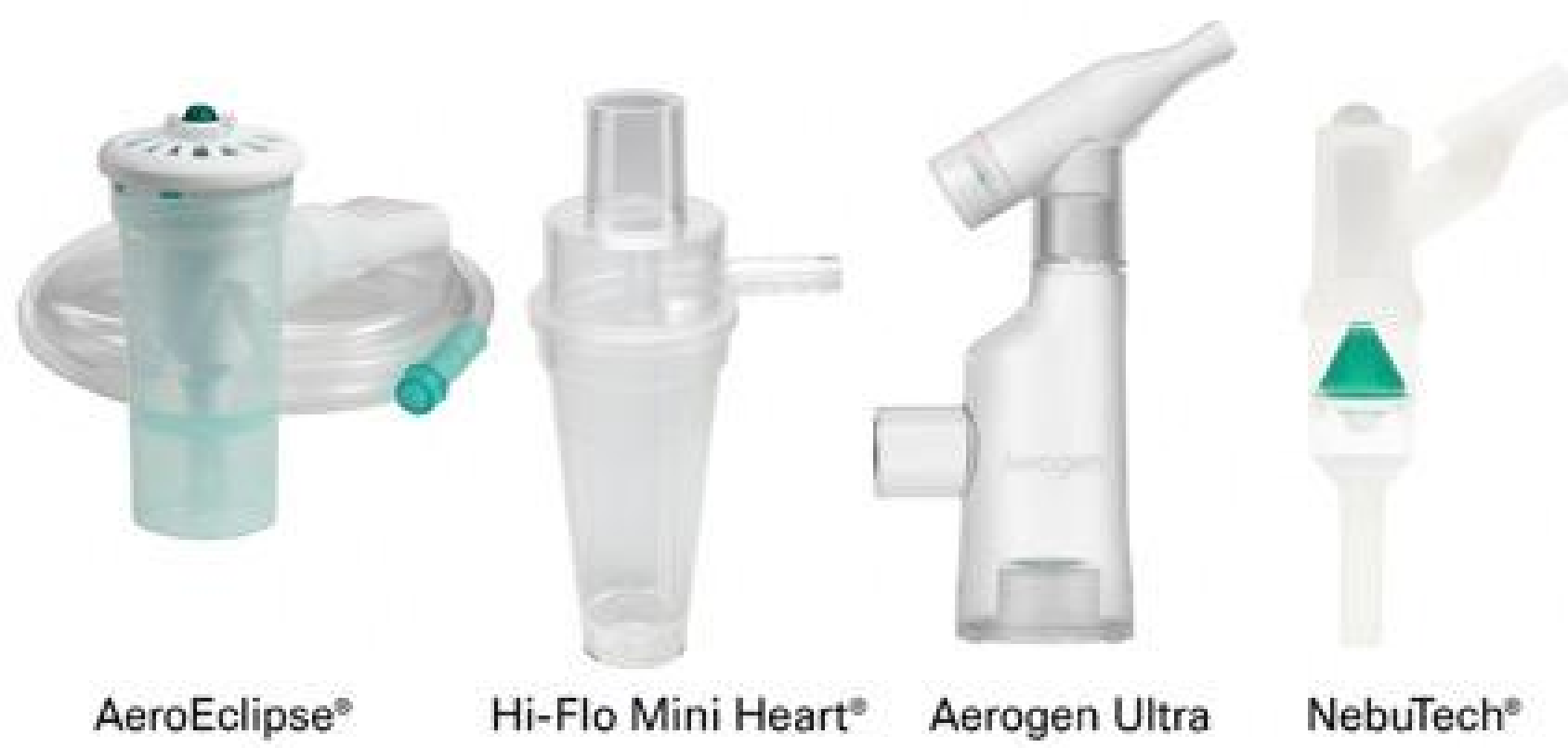

Supplement: Supplementary file 1 [file wjem-27-1611-s001.pdf]
